# Mating changes the female dietary preference in the two-spotted cricket, *Gryllus bimaculatus*

**DOI:** 10.3389/fphys.2014.00095

**Published:** 2014-03-13

**Authors:** Yusuke Tsukamoto, Hiroshi Kataoka, Hiromichi Nagasawa, Shinji Nagata

**Affiliations:** ^1^Department of Integrated Biosciences, Graduate School of Frontier Sciences, The University of TokyoChiba, Japan; ^2^Department of Applied Biological Chemistry, Graduate School of Agricultural and Life Sciences, The University of TokyoTokyo, Japan

**Keywords:** feeding behavior, geometric framework, *Gryllus bimaculatus*, post-mating behavior

## Abstract

Most insect species exhibit characteristic behavioral changes after mating. Typical post-mating behaviors in female insects include noticeable increases in food intake, elevated oviposition rates, lowered receptivity to courting males, and enhanced immune response. Although it has been reported that mated females of several insect species including the fruit fly, *Drosophila melanogaster* increase the amount of food intake and change their dietary preferences, the limited number of comparative studies prevent the formulation of generalities regarding post-mating behaviors in other insects in particular amongst orthopteran species. Here, we investigated whether females of the two-spotted cricket, *Gryllus bimaculatus*, alter their feeding behavior after mating. Although significant differences in the amount of food intake after mating were not observed, all experimental data indicated a clear trend among crickets toward the ingestion of larger quantities of food. Geometric framework analyses revealed that the mated female crickets preferred food with higher protein content compared to virgin female crickets. This implies that this species required different nutritional demands after mating. These findings further expand our understanding of the behavioral and biological changes that are triggered in female insects post-mating, and highlight the potential for this species in investigating the molecular-based nutritional dependent activities that are linked to post-mating behaviors.

## Introduction

Most animals, including insects, exhibit a repertoire of behavioral and physiological changes after mating. The characteristic changes in the post-mating behavior in insects have been extensively studied and reported (Wolfner et al., 2005). In general, it has been demonstrated that insects exhibit major behavioral changes immediately after mating, with this phenomenon being more exaggerated in the mated females compared to the mated males. The mated females of the fruit fly, *Drosophila melanogaster*, exhibit a number of characteristic post-mating behavioral changes such as the stimulation of ovulation and reduction of receptivity for courting males (Wolfner, 1997). In addition, the female immune system is activated in responses to male seminal fluid factors (Lawniczak and Begun, 2004; McGraw et al., 2004; Peng et al., 2005). Similar behavioral changes related to reproduction have also been observed in other insect species, including the common house mosquito, *Culex pipiens* (Chiba et al., 1992), the Mediterranean fruit fly, *Ceratitis capitata* (Jang, 2002), in lepidopteran species, such as the cotton bollworm, *Helicoverpa armigera* (Jin and Gong, 2001), in hemipteran species, such as the western tarnished plant bug, *Lygus hesperus* (Brent, 2010) and in coleopteran species, such as the ground beetle *Leptocarabus procerulus* (Takami et al., 2008).

Of the various recorded characteristic post-mating behaviors, motivated feeding behavior has also been reported in several insects (Table 1). For example, after mating, *D. melanogaster* females increase the amount of food intake (Carvalho et al., 2006). Similarly, motivated feeding behavior has been observed across several insect orders; the two-spotted ladybird, *Adalia bipunctata* (Perry, 2011) in Coleoptera, the yellow fever mosquito, *Aedes aegypti* (Judson, 1967) in Diptera and *L. hesperus* (Cooper and Spurgeon, 2011) in Hemiptera. It has also been demonstrated that the mated females of *D. melanogaster* alter dietary preference (Ribeiro and Dickson, 2010; Vargas et al., 2010). It is likely that the post-mating events including copulation, ejaculation, and oogenesis motivated their feeding and changes the feeding preference in several insect species. Although a number of physiological studies have been performed concerning post-mating feeding behavior, molecular-based studies remain limited.

**Table 1:** **Post-mating feeding behavior changes in several insect species**.

**Order**	**Species**	**Post-mating feeding behavioral changes**	**References**
Diptera	*Drosophila melanogaster*	Increasing the amount of food intake	Carvalho et al., 2006
		Increasing the preference of yeast	Ribeiro and Dickson, 2010; Vargas et al., 2010
	*Aedes aegypti*	Altered the feeding pattern	Judson, 1967
		Increasing the amount of blood meal intake	Adlakha and Pillai, 1976
	*Glossina austeni*	Increasing the amount of blood meal intake	Ejezie and Davey, 1977
	*Culex pipiens*	Increasing the amount of blood meal intake	Adlakha and Pillai, 1976
Coleoptera	*Adalia bipunctata*	Increasing the amount of food intake	Perry, 2011
Hemiptera	*Lygus hesperus*	Spending more time for stylet-probing	Cooper and Spurgeon, 2011

To date, RNA interference techniques and genomic editing manipulations have proven to be powerful tools for exploring numerous biological phenomena at the molecular level, even in non-model insect species such as the two-spotted cricket, *Gryllus bimaculatus* (Nakamura et al., 2010; Konuma et al., 2012; Watanabe et al., 2012). Based on previous studies, it has become increasingly evident that the two-spotted cricket represents a good model species for studying the post-mating effects on feeding behavior.

In this study, we investigated whether *G. bimaculatus* follows the pattern established in other insects regarding the effects of mating on feeding behavior. To address the intriguing issues, we performed several physiological experiments to monitor the feeding behavioral change in adult female *G. bimaculatus* after mating. We specifically focused on different feeding parameters, including the amount of food intake and dietary preference.

## Materials and methods

### Chemicals and reagents

Cellulose powder used in the present study was purchased from Nacalai tesk (Kyoto, Japan). Dextrin hydrate and casein were purchased from Wako Pure Chemical Industries (Osaka, Japan). For cricket food, rabbit ORC4 was purchased from ORIENTAL YEAST Co., Ltd. (Tokyo, Japan) and a commercially available diet of cat food (Friskies dry mix) was purchased from Nestle Purina PetCare Co., Ltd. (Hyogo, Japan).

### Insects

Fifth instar larvae of the two-spotted cricket, *G. bimaculatus*, were purchased from Tsukiyono Farm Co., Ltd. (Gunma, Japan). Crickets were reared in plastic containers (55 × 39 × 31 cm) at 27 ± 1°C with 70 ± 5% relative humidity under long-day lighting conditions (16-h light, 8-h dark cycle). Crickets were fed *ad libitum* on a mixture of rabbit ORC4 and cat food in a ratio of approximately one to one with unlimited access to water. Although most crickets grow and molt simultaneously after arriving at the laboratory, we used crickets with synchronous growth from the last instar to adult emergence. Because the amount of food intake for the first 5 days after adult emergence was remarkably inconstant, we used virgin females 7–10 days after adult emergence. Male crickets used in this study were reared with adult females.

### Measurement of the amount of food intake

To measure the amount of food intake, we prepared “commercial” diet tablets made of cellulose powder, rabbit ORC4, and dextrin. Powdered rabbit ORC4 and dextrin were mixed with cellulose powder; rabbit ORC4:dextrin = 2:1 (wt/wt). Cellulose powder was added to the mixture at a final concentration of 70%. The mixture was transferred into a plastic template (φ2.1 × 0.5 cm). The mixture was hardened after wetting with water by pushing under hand-pressure, and then frozen at −80°C. The frozen tablets were lyophilized to dryness. The resulting tablets were weighed as starting test diets. Before measuring the weight of the remaining tablets after feeding, the water in the tablets was removed by heating at 70°C for 3 h. Adult virgin females fed *ad libitum* were isolated individually in round plastic containers (φ9.9 × 9.4 × 4.5 cm) and acclimated to the synthetic tablet diets for 1 day before experimental treatment. In assays measuring the amount of food intake, adult females kept isolated in the containers were examined for 3 days after acclimation. As the different periods (for 1, 2, and 3 days) of acclimation for dietary tablet do not change the trends of the results, we acclimated for 1 day in the present study. The mated females were prepared by putting adult male crickets into containers with a virgin female. After the end of mating, the males were removed, and then diet tablets were given to the mated females. As the remaining tablets were removed to measure the amount of food intake, new tablets were supplied every day. During this experiment, crickets could freely access water.

### Food choice test

To test the dietary preference of the crickets, we prepared two different “generic” diet tablets made of cellulose powder, dextrin, and casein. Dextrin and casein were mixed with cellulose powder at different ratios by weight: Carbohydrate-rich diet—dextrin: casein (10:1); Protein-rich diet—dextrin: casein (1: 10). Each tablet was adjusted with cellulose powder to a final concentration of 67%. Adult virgin females fed *ad libitum* were isolated in the round plastic containers and acclimated to the two different diet tablets with free access to water for 1 day before the experimental treatment. After that, adult male crickets were transferred into containers for mating. After the end of mating, the males were removed, and then diet tablets were given to the mated females. As the remaining tablets were removed to measure the amount of food intake, new tablets were supplied every day. Geometric analyses were performed based on the previous description (Raubenheimer and Simpson, 1993; Simpson and Raubenheimer, 1995).

### Statical analyses

Comparisons across two groups were statistically analyzed by Student's *t*-test and Dunnett's test. Comparison of food intake in individuals was analyzed by paired *t-*test. *P*-values less than 0.05 were considered to be statistically significant. All experiments were performed at least twice and the authors confirmed that the resulting data were reproducible.

## Results

### Amount of food intake after mating in *G. bimaculatus*

**Figure 1:**
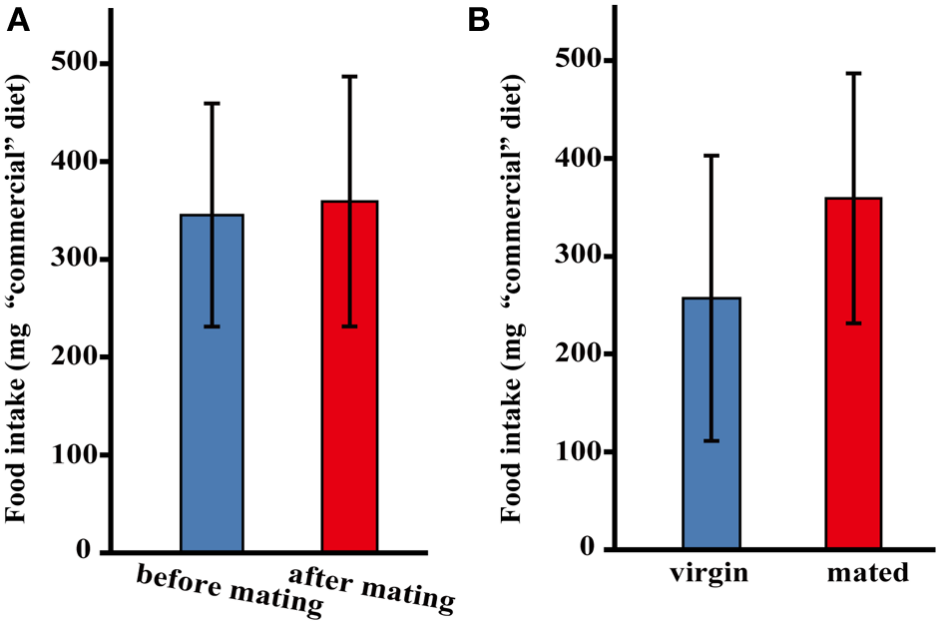
**The amount of “commercial” diet intake after mating in *G. bimaculatus* females**. The amount of food intake by *G. bimaculatus* adult females after mating was measured by consumption of “commercial” diet tablets. **(A)** The amount of food intake by individual females before mating was also measured as a control. *N* = 8, Mean ± *SD*. Data was statistically analyzed using a paired *t-*test. The amount of food intake by females before and after mating was compared. **(B)** The amount of food intake by the mated females was measured by comparing to the amounts of food intake by the virgin females. Mated females; *N* = 8, virgin females; *N* = 10, Mean ± *SD*. Data was statistically analyzed using Dunnett's test.

Throughout our observations of the adult female two-spotted cricket, *G. bimaculatus* under our rearing conditions, we noticed that, similar to other insect species, the mated females appeared to increase their food intake (Table 1). To confirm if this species will undergo a change in post-mating feeding behavior; we first measured the amount of food intake by mating in the female adult crickets using “commercial” dietary tablets made of rabbit ORC4, dextrin and cellulose powder. Observation revealed that the amount of food intake in adult females before mating was 345 ± 113.6 mg/day, while that in adult females after mating was 359 ± 127.6 mg/day; a statistically significant increase in the amount of food intake was not found (Figure 1A). On the other hand, the mated females ingested more food (359 ± 127.6 mg/day) compared to control virgin females (257 ± 145.7 mg/day); but not significantly (Figure 1B).

### Changes in dietary preference after mating

**Figure 2:**
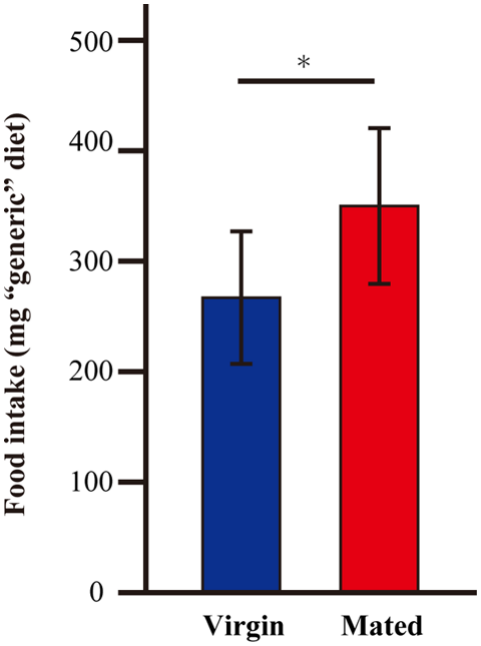
**The amount of “generic” diet intake after mating in *G. bimaculatus* females**. The amount of food intake by *G. bimaculatus* adult females after mating was measured by consumption of two different “generic” diet tablets: carbohydrate-rich and protein-rich tablets. The amount of food intake by the mated females was measured and was compared with the amount of food intake by the virgin females. *N* = 7, Mean ± *SD*. Data was statistically analyzed by Student's *t-*test. *P*-values less than 0.05 were considered to be statistically significant. ^*^P < 0.05.

**Figure 3:**
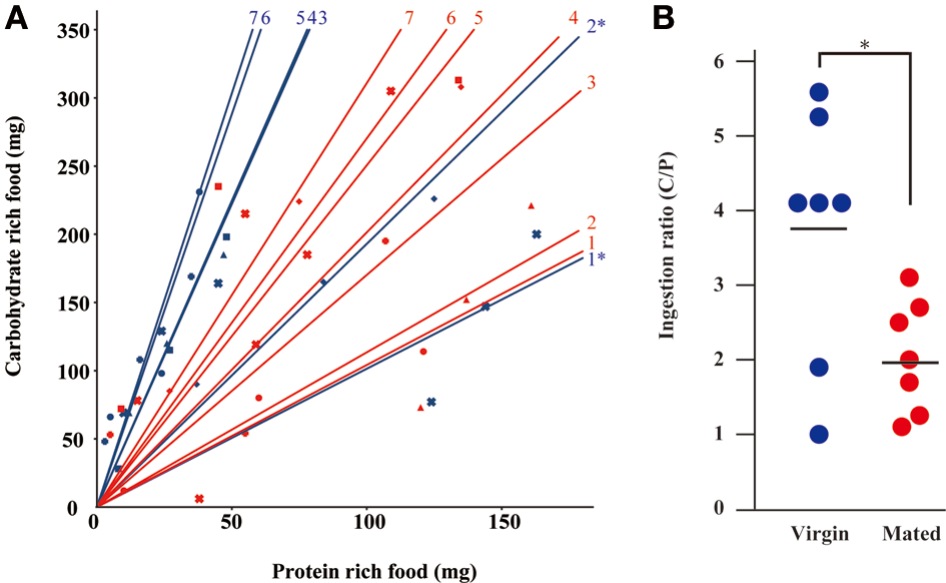
**The transition of dietary preference after mating. (A)** The geometric framework analysis by mated female crickets. The horizontal axis represents the amount of protein-rich food intake and the vertical axis represents the amount of carbohydrate-rich food intake using “generic” diet tablets. Red and blue spots indicate the measured values from mated and virgin females, respectively. Different symbols indicate different individuals. Red and blue approximate lines indicate mated and virgin females, respectively. Data were plotted as every day. The same symbol indicates the points of the amount of food ingested each day individual. Each data point was plotted as an accumulated value from the first observation day in this figure. The numbers of individuals examined are indicated. An asterisk represents the data from an unusual cricket as described in the text. **(B)** The ingestion ratio of “generic” casein-rich diet/protein-rich diet (C/P) by mated and virgin females. Dots indicate the data from individual crickets. Bars indicate mean values. Data were statistically analyzed by Student's *t-*test. *P-*values less than 0.05 were considered to be statistically significant. ^*^P < 0.05. *N* = 7, Mean ± *SD*.

**Figure 4:**
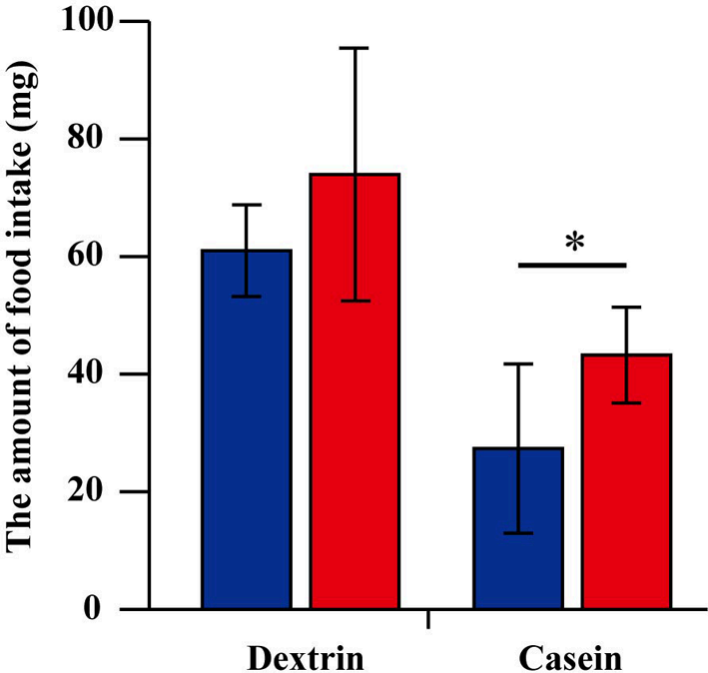
**The amount of dextrin and casein consumption**. The amount of dextrin and casein consumption. The value is computed using the amount of consumption of carbohydrate-rich and protein-rich “generic” diet tablets and the rate of the nutrients contained in each “generic” tablet. The amount of dextrin consumption is estimated by summing up 30% of the consumed amount of carbohydrate-rich tablet and 3% of the consumed amount of protein-rich tablet. The amount of casein consumption is estimated by summing up 30% of the consumed amount of protein-rich tablet and 3% of the consumed amount of carbohydrate-rich tablet. *N* = 7, Mean ± *SD*. Data were statistically analyzed by Student's *t-*test. *P-*values less than 0.05 were considered to be statistically significant. ^*^P < 0.05.

Because *G. bimaculatus* did not show a significant increase of food intake after mating, we sought to explore the details of the slight increase of food intake. We examined whether mated females alter their dietary preference. Mated females were subjected to a choice assay for 3 days using two nutritionally different “generic” diet tablets (carbohydrate-rich and protein-rich diet tablets). Unlike the first experiment (Figures 1A,B), the mated females ate significantly more than virgins when carbohydrate-rich and protein-rich diet tablets were supplied (Figure 2). To evaluate the effects of mating on the dietary preference of adult females, we conducted a geometric framework analysis, which has been frequently used for analyses of dietary preferences (Raubenheimer and Simpson, 1993; Simpson and Raubenheimer, 1995). Mated females were fed *ad libitum* on both of the tablets for 3 days. The geometric framework analyses by using data from individual crickets indicated that mated females preferred protein-rich diet tablets to carbohydrate-rich tablets compared to the virgin females (Figures 3A,B). However, a number of individuals exhibited distinctive trends in preference from the major population (asterisks in Figure 3A). Even though these individuals ruled out the tendency of preference, we were able to obtain significant differences in different experimental trials. To confirm if mated females exhibit motivated feeding on a protein-rich diet, we next examined the amount of each nutrient consumed. The mated females ate as much dextrin as the virgin females (Figure 4), while mated females ate approximately twice the amount of casein as virgin females (Figure 4).

## Discussion

In the present study, we demonstrated that immediately after mating the adult female did not exhibit a significant increase in the amount of food intake compared to virgin females when tablets made of rabbit ORC4 were used. This contrasts with previous studies on other insect, such as *D. melanogaster* (Carvalho et al., 2006) and *A. bipunctata* (Perry, 2011), which reported significant increases in food intake after mating. Although our result was not statistically significant, the similar tendency to increase the amount of food intake was observed in at least five trials of observation. These unexpected results might be caused by using diet tablets made of rabbit ORC4, which is a commercially available artificial diet that contains several nutrients. ORC4 for phytophagous animals might not be as sufficient for omnivorous crickets in satisfying their nutritional demands. In fact, a commercially available cat food provided different data albeit with a similar tendency to motivate feeding after mating. On the other hand, when tablets that contained dextrin and casein as nutrients were used, the amount ingested by mated females increased (Figure 2). These results suggest that the amount of food intake depends on the nutritional contents of the dietary source.

The selection of diet are not only due to the nutritional states but also due to the nutritional contents of food as the previous report also suggested in other insect species. The elicited preference in protein-rich food by mated females (Figures 3A,B) is reminiscent of that observed in the post-mated *D. melanogaster* females, in which mated females ate more yeast compared to virgin females (Ribeiro and Dickson, 2010; Vargas et al., 2010). If the mated *D. melanogaster* adult females consumed yeast as a protein or nitrogen source, it is consistent with the data obtained by the present observations. This hypothesis is also supported by other recent studies suggesting that protein from the ingested diet by *D. melanogaster* adult females is used as nitrogen source for producing eggs (Tatar, 2011). Therefore, the elicited protein preference after mating might be conserved across insect species, with protein being an essential nutrient for oogenesis and oviposition. In preliminary observations, crickets laid eggs from the first day after mating to early on the second day (data not shown). In contrast, preferential feeding on the high protein diet started to increase late on the second day (Figure 3A). These observations suggest that egg laying is differentially timed to the initiation of protein food preference after mating. Therefore, it is possible that egg laying is strongly linked to the change in dietary preference. *G. bimaculatus* females are able to lay eggs multiple times in their lives. Perhaps, the different amounts of dietary protein ingested by mated females compared to virgin females is directly linked to the demands for generation of the newly developing eggs for subsequent mating and egg laying events.

It is intriguing that imbalanced nutritional states might potentially cause a specific nutritional dependent feeding behavior, as observed in the mated females in this study, and other animal species in previous studies. In the case of post-mating behavior, this change in a possible nutritional state may result from seminal factors penetrating through the ovarian barrier layer (Avila et al., 2011). It has been observed in many insect species that a number of seminal fluid factors influence post-mating events (Gillott, 2003; Avila et al., 2011). For example, the 36 amino acid accessory gland derived *D. melanogaster* sex peptide (SP) is a striking example of a factor influencing mating-activated events (Chen et al., 1988; Kubli and Bopp, 2012). This peptide penetrates through the ovarian layer in the mated females from the ejaculated seminal fluid (Pilpel et al., 2008). Genetic manipulation of SP-related genes has shown that SP causes some changes in the feeding behavior of adult female *D. melanogaster* immediately after mating (Kubli and Bopp, 2012). In addition, SP-like peptides have also been found in some insect species such as *Helicoverpa armigera* (Nagalakshmi et al., 2007). In *H. armigera* an SP-like peptide and its receptor mediate pheromone biosynthesis and re-mating rejection behavior against courting males (Nagalakshmi et al., 2007; Hanin et al., 2011). SP and SP-like peptides are not conserved across insect species. Although SP is not present in some insect species, the SP receptor is highly conserved (Yapici et al., 2008; Kim et al., 2010; Poels et al., 2010). Therefore, further research is required to elucidate whether changes in feeding behavior after mating are directly mediated by SP and SPR mechanisms. The lack of a sequenced *G. bimaculatus* genome makes it difficult to ascertain if SP-like peptides and SPR are present in *G. bimaculatus*. First, it will be particularly important to confirm and characterize the contribution of seminal factors, including SP and SPR, possibly contributing to changes in the feeding behavior of *G. bimaculatus*, and to determine the mechanisms by which such dramatic changes in the post-mating behavior are affected.

It is interesting that the transition of nutritional requirements during the transient post-mating period is similar to “self-selection” which is a crucial and fundamental behavior for polyphagous and oligophagous animals to recognize and compensate for their insufficient nutrients from their selected diets (Richter et al., 1938). This self-selection is observed in many animals, including insects (Waldbauer and Friedman, 1991). Therefore, animals are able to select suitable nutrients to fit their dietary demands when feeding *ad libitum*. As observed in this study, mated female crickets preferentially selected the protein-rich diet. This behavior indicates that mated crickets shift their nutritional demands from a normal state to a specific state where they are able to select the required nutrients.

## Conclusion

Mated female *G. bimaculatus* crickets tended to eat more food compared to virgin females. In addition, geometric framework analyses revealed that *G. bimaculatus* females exhibited a transient switch to a protein rich diet immediately after mating.

### Conflict of interest statement

The authors declare that the research was conducted in the absence of any commercial or financial relationships that could be construed as a potential conflict of interest.
